# The 12-item WHO Disability Assessment Schedule II as an outcome measure for treatment of common mental disorders

**DOI:** 10.1017/gmh.2016.7

**Published:** 2016-04-18

**Authors:** C.C. Thomas, S.D. Rathod, M.J. De Silva, H.A. Weiss, V. Patel

**Affiliations:** 1 Department of Psychology, Princeton University, Princeton, NJ, USA; 2 Department of Population Health, London School of Hygiene and Tropical Medicine, London, UK; 3 MRC Tropical Epidemiology Group, London School of Hygiene and Tropical Medicine, London, UK; 4 Sangath, Alto-Porvorim, Goa, India

**Keywords:** 12-item WHODAS, anxiety, depression, disability, etiology, validity

## Abstract

**Background.:**

Common mental disorders (CMD) are among the most significant contributors to disability worldwide. Patient-reported disability outcomes should be included as a key metric in the comparative assessment of value across global mental health interventions. This study aims to evaluate the validity of a widely used, cross-cultural tool – the 12-item World Health Organization Disability Assessment Schedule II (WHODAS) – as a functional outcome measure for CMD treatment.

**Methods.:**

The study population includes 1024 participants with CMD enrolled in the MANAS trial in India. CMD was assessed using the Revised Clinical Interview Schedule (CIS-R). Disability was assessed using the 12-item WHODAS II plus a measure of disability days. This analysis presents the correlations between these disability items and CMD symptom severity at 2 months after enrollment (convergent validity) and the items’ associations with CMD recovery 4 months later (external responsiveness).

**Results.:**

All items showed a positive correlation of disability with CMD symptom severity (*p* < 0.001). The WHODAS items of ‘standing,’ ‘household responsibilities,’ and ‘emotional disturbance’ explained the most variance in CMD symptom severity. Improvements in ‘disability days,’ ‘emotional disturbance,’ ‘standing,’ ‘household responsibilities,’ ‘day-to-day work,’ and ‘concentrating’ were significantly associated with CMD recovery over follow-up.

**Conclusions.:**

Further research is recommended on a CMD-specific WHODAS subscale comprised of the six WHODAS items found to be most strongly associated with CMD severity and recovery. This shorter, CMD-specific disability subscale would critically serve as a common metric to compare intervention impact on patient-centered outcomes and, in turn, to allocate global mental health resources efficiently.

## Background

Common mental disorders (CMD), comprising depressive and anxiety disorders, cause significant disability worldwide, with depressive disorders alone estimated to be the second leading global contributor (Ferrari *et al.* 2013). CMD are associated with such disabling consequences as diminished economic productivity, loss of employment, and impaired social functioning and, overall, contribute substantial socioeconomic burdens on patients and communities (Ormel *et al*. 1994; Judd *et al.* 2000; Patel *et al.* 2010; Silva *et al.* 2013). In part due to the large burden of CMD globally, particularly in low- and middle-income countries with wide treatment gaps, the regular assessment of disability outcomes of mental health interventions has been selected as one of the top 25 research priorities for the field of global mental health through a Delphi panel of hundreds of international stakeholders (Collins *et al.* 2011).

Disability is defined by the World Health Organization (WHO) as the disruption of an individual's interaction with his or her environment (WHO, 2010). A common metric of self-reported disability is a necessary tool for the evaluation and comparison of intervention impact on outcomes that matter to patients. While the diversity and reach of psychosocial treatments for common mental disorders have been rapidly growing over the past several decades, a recent systematic search found that fewer than 5% of clinical trials for the treatment of depression reported a measure of functional outcomes (McKnight & Kashdan, 2009). In the burgeoning movement that extends and tailors mental health treatments to new cultures and contexts, there is a need for a common metric for mental health program evaluation that includes patient-centered outcomes.

Due to its widespread international use, sound psychometric properties, and ease of administration, the WHO Disability Assessment Schedule II (WHODAS) is a prime candidate for the routine evaluation of intervention impact on patient-reported disability outcomes (WHO, 2010). Developed in 1998, the WHODAS is a cross-cultural tool that captures social, occupational, physical, and role impairments associated with a health condition (WHO, 2010). As a component in disease burden calculations (i.e. through Disability-Adjusted Life Years), the WHODAS enables the comparison of cost-effectiveness across diverse health interventions, which in turn guides planning of health policies and programs. While the WHODAS has heretofore been used primarily to assess disability associated with physical illnesses, the WHODAS is being increasingly applied to study disability among psychiatric populations in low- and middle-income countries. For example, it has been applied to assess functional impairments associated with mental disorders, understand relationships between mental illness and physical comorbidities, and measure mental health treatment outcomes (Akinsulore *et al.* 2015, De Silva *et al.* 2015, Faye *et al.* 2015).

This study aims to take the first step in evaluating the WHODAS as an outcome measure for CMD treatment by assessing the convergent validity of WHODAS disability items with CMD severity and the external responsiveness of items to recovery from CMD. The individual WHODAS items found to be more correlated to CMD severity and responsive to CMD recovery are furthermore recommended as candidates for future development of a CMD-specific WHODAS subscale. This subscale would strengthen assessment of functional outcomes in studies of CMD recovery.

## Methods

### Study sample

The study sample for this secondary analysis includes patients enrolled in a cluster randomized controlled trial of CMD treatments in Goa, India (the MANAS trial) (Chatterjee *et al*., 2008; Patel *et al*. 2010, 2011). This sample includes patients diagnosed with CMD, according to the International Statistical Classification of Diseases and Related Health Problems – 10th revision (ICD-10), being treated in the collaborative stepped-care (*n* = 423) and enhanced usual care (*n* = 601) arms. Conducted between 2007 and 2009, the MANAS trial included 2796 adult primary care attenders who screened positive for CMD according to the 12-item General Health Questionnaire (GHQ) in 12 public health centers and 12 private general practitioner clinics. Details of the study design, the baseline characteristics of participants, and the treatments effects on CMD and disability outcomes have been published elsewhere (Chatterjee *et al.* 2008; Patel *et al.* 2008, 2010, 2011). The MANAS trial is registered with ClinicalTrials.gov with identifier NCT00445407.

### Study design

Disability measurements were taken at the 2-, 6-, and 12-month time points after enrollment in the MANAS trial. Of the 2491 participants attending the 2-month follow-up, 1024 (41.1%) maintained a diagnosis of CMD. This subsample of 1024 participants was included in the current analysis as a cohort followed from 2 to 6 months after enrollment.

### Instruments

Clinical outcomes were measured with the Revised Clinical Interview Schedule (CIS-R), a standardized, structured CMD diagnostic tool that has been field-tested for use in Goa (Lewis *et al.* 1992; Patel *et al.* 1998, 2011). Lay health workers used the CIS-R to generate an ICD-10 diagnosis and a symptom severity score (0–57) at each follow-up point (Patel *et al.* 2010). Recovery from CMD at the 6-month follow-up visit was determined with the CIS-R according to ICD-10 criteria (Patel *et al.* 2010).

Disability outcomes were assessed using the 12-item WHODAS II, and two items from the 36-item WHODAS II. The WHODAS II measures items in six domains of functioning as experienced over the past 30 days: mobility, self-care, life activities, understanding and communicating (U&C), interpersonal interactions, and participation in society (WHO, 2010). The 12-item tool assesses each domain with two items that are measured on a 3-point scale in which 1 indicates no disability, 2 indicates mild to moderate disability, and 3 indicates severe to extreme disability. These items were summed to generate a total score between 12 (no disability) and 36 (maximum disability). A total number of disability days were computed from two items of the 36-item WHODAS assessing days of no work or of reduced work due to illness in the past 30 days and was assessed by lay health workers if patients reported at least mild disability on any of the 12 WHODAS items (Patel *et al.* 2008).

### Statistical analyses

The convergent validity of each WHODAS item with the CIS-R symptom severity score was evaluated using a non-parametric equivalent of the Pearson's correlation coefficient, Spearman's rank-order correlation coefficient *r*_s_, which indicates the extent of correlation between the disability item score and the CIS-R score. Similar to squaring Pearson's correlation coefficient, Spearman's rank-order correlation coefficient may be squared (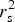) to estimate the variance in CIS-R scores explained by the disability item score as an effect size statistic (Rosenthal, 1994; Pett, 1997). Qualitative evaluations were used to assess the degree of correlation, with Pearson's correlation values of approximately 0 (no correlation to weak correlation), ±0.50 (moderate correlation), ±0.71 (high correlation), and ±1 (perfect correlation) corresponding to Spearman's rank correlations of 0, ±0.48, ±0.69 and ±1 (Kirkwood & Sterne, 2003).

To identify which specific aspects of disability were most responsive to recovery, we used logistic regression to estimate magnitude of the relationship between change in individual WHODAS items and the CMD recovery outcome (i.e. external responsiveness). Recovery from CMD between the 2- and 6-month follow-up points was analyzed as the dependent variable. The change scores for each of the 12 WHODAS items, the global WHODAS score, and the total number of disability days were coded into binary measures of 0 = ‘not improved’ and 1 = ‘improved’. Each WHODAS measure was analyzed as an independent variable in separate logistic regression models, controlled for baseline CIS-R score, sex, age, clinic type, and intervention arm as potential confounders. Cluster robust standard errors (s.e.) were applied to adjust the 95% confidence intervals (CI) for clinic-level clustering.

## Results

### Sample characteristics

The sociodemographic and clinical characteristics of the sample at the 2-month follow-up are presented in Table 1. They had a mean age of 48.0 years (s.d. = 13.6) and were predominantly female (85.8%), married (61.7%), and Hindu (70.2%). Half had no formal education (50.0%), and a majority was unemployed (71.5%). The median CIS-R score was 18 out of 57 (IQR: 14–24), the median WHODAS score 19 out of 36 (IQR: 16–22), and the median disability days score 15 out of 30 (IQR: 0–30). Of the initial 1024 participants, 943 (92.1%) were seen at 6 months, and of these, 368 participants (39.0%) had recovered from CMD. At both 2 and 6 months, respectively, only four patients (0.4%) had missing values for the disability days score.

**Table 1.** Distribution of study sample characteristics at 2 months after recruitment into the MANAS trial, India, 2007–2009

Sociodemographic characteristics		Mean (s.d.)/number (%)
Age (*n* = 1024)		48.0 (13.6)
Sex (*n* = 1024)	Female	878 (85.8)
	Male	146 (14.2)	
Education level (*n* = 1024)	<1 year	512 (50.0)
	1–5 years	230 (22.5)
	≥6 years	282 (27.5)
Employment (*n* = 1023)	Unemployed	731 (71.5)
	Full-time	126 (12.3)
	Part-time/seasonal	150 (14.7)
	Other	16 (1.6)
Marital status (*n* = 1024)	Married	632 (61.7)
	Widowed or separated/divorced	339 (33.1)
	Never married	53 (5.2)
Ethnicity (*n* = 1024)	Goan	974 (95.1)
	Other	50 (4.9)	
Religion (*n* = 1024)	Hindu	719 (70.2)
	Christian	288 (28.1)
	Muslim	16 (1.6)
Financial status (*n* = 1024)	Living comfortably	64 (6.3)
	Just getting by	361 (35.3)
	Difficult to make ends meet	599 (58.5)
Clinical characteristics		Median (IQR; range)
CIS-R score (*n* = 1024)		18 (14–24; 0–57)
Twelve-item WHODAS II global score (*n* = 1024)		19 (16–22; 12–36)
Disability days score (*n* = 965)		15 (0–30; 0–30)

### Convergent Validity

**Fig. 1.**
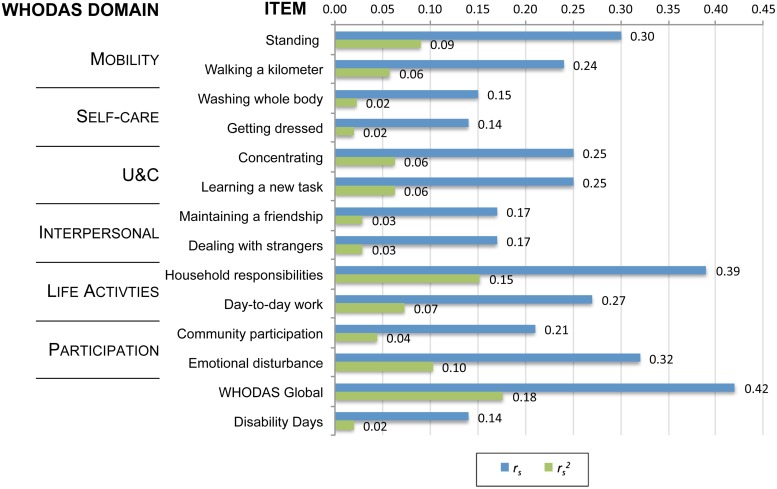
Convergent validity of CMD symptom severity scores with WHODAS disability measures at 2 months among participants of the MANAS trial, India, 2007–2009.

Convergent validity statistics are presented in Fig. 1. All of the disability items had weak but significant correlations with CIS-R scores, with *r*_s_ values between 0.14 and 0.39. ‘Household responsibilities,’ ‘emotional disturbance,’ and ‘standing’ explained the most variance in CIS-R scores (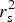 values of 0.15, 0.10, and 0.09, respectively). The WHODAS global score showed the greatest extent of correlation with the CIS-R scores (*r*_s_ = 0.42), yet remained only moderate, explaining 18% of the variance in CIS-R scores (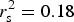).

### External Responsiveness

As presented in Table 2, improvement in the WHODAS global score, disability days score, and five of the 12 WHODAS item scores were associated with CMD recovery. Participants whose global WHODAS scores improved between 2 and 6 months follow-up had on average 2.40 times higher odds of having recovered from CMD [adjusted Odds Ratio (aOR) 95% CI: 1.62–3.56]. Those who reported fewer disability days at 6 months had 2.37 higher odds of recovery (aOR 95% CI: 1.66–3.37). Of the 12 individual WHODAS items, five displayed evidence of significant responsiveness to CMD recovery: ‘emotional disturbance,’ ‘standing,’ ‘household responsibilities,’ ‘day-to-day work,’ and ‘concentrating.’

**Table 2.** External responsiveness of improvements in disability items with CMD recovery at 6 months among participants of the MANAS trial, India, 2007–2009

	Domain	Item	Adjusted OR (95% CI)
12-item WHODAS II (*n* = 943)	Mobility	Standing	1.67 (1.19–2.34)***
		Walking a kilometer	1.23 (0.87–1.72)
	Self-care	Washing whole body	1.10 (0.69–1.75)
		Getting dressed	0.83 (0.51–1.34)
	Understanding and communicating	Concentrating	1.30 (1.01–1.67)**
		Learning a new task	0.99 (0.70–1.40)
	Inter-personal	Maintaining a friendship	0.76 (0.36–1.58)
		Dealing with strangers	0.94 (0.52–1.69)
	Life activities	Household responsibilities	1.67 (1.19–2.34)***
		Day-to-day work	1.40 (1.01–1.94)**
	Participation	Community participation	1.03 (0.70–1.50)
		Emotional disturbance	1.81 (1.27–2.58)***
	Global score		2.40 (1.62–3.56)***
Disability days in past month (*n* = 836)			2.37 (1.66–3.37)***

Odds ratios adjusted for baseline CIS-R score, sex, age, clinic type, and intervention arm, with cluster robust standard errors (s.e.).

* *p* < 0.1, ** *p* < 0.05, *** *p* < 0.01, *p* value from Wald test in fully adjusted model.

## Conclusions

This study constitutes, to our knowledge, the first assessment of the validity of the 12-item WHODAS II among middle-aged patients with CMD in a low- or middle-income country. Previous studies have analyzed associations between measures of disability and CMD at a single time point or changes in disability over time as estimates of internal responsiveness without reference to CMD caseness (Judd *et al.* 2000; Garin *et al.* 2010). As a longitudinal treatment study, this study importantly adds quantifications of the associations between concurrent changes in disability and CMD caseness to the evidence base.

The present study found that five items in the domains of participation (‘emotional disturbance’), physical mobility (‘standing’), life activities (‘household responsibilities’ and ‘day-to-day work’), and understanding and communicating (‘concentrating’) most strongly correlated with symptom severity in the cross-section and responded to CMD recovery. In contrast, items in the interpersonal and self-care domains showed little to no relationship with severity or recovery. Despite weak convergent validity, the disability days score showed strong responsiveness to recovery. Although correlation coefficients for all WHODAS items with CIS-R scores were weak, five WHODAS items and the disability days item showed the greatest extent of correlation with CMD severity and significant associations with CMD recovery; thus, these six WHODAS items are recommended for further research in the development of a CMD-specific subscale.

This pattern of results largely aligns with studies of patients with depression in Europe and the USA in which the WHODAS domains of participation and life activities have been found to be the most internally responsive (i.e. change over time) (Chwastiak & Von Korff, 2003; Garin *et al*. 2010). In contrast to Chwastiak & Von Korff (2003), this study found the self-care domain to be responsive and the interpersonal domain to be largely unresponsive. However, in this study few patients with CMD experienced disability in this domain at 2 months, and it is possible that gains in interpersonal functioning occurred largely within the first 2 months of the trial.

Unlike much of the research on patients with depression in high-income countries, physical mobility items showed strong responsiveness. A qualitative study of the MANAS trial found weakness/tiredness to be one of the two most commonly reported reasons for seeking care, suggesting a somatic manifestation of CMD in this context (Andrew *et al.* 2012). Thus, the physical mobility domain may be heterogeneously associated with CMD cross-culturally, and the relationship may differ for those with anxiety versus those with depressive disorders.

The observed external responsiveness of the disability days measure to CMD recovery is consistent with findings from a review of depression treatment studies in high-income settings. Notably, Mintz *et al.* (1992) found that work role impairments, including disability days, were more consistently alleviated when patients’ symptoms were also alleviated and, moreover, that most of the variance in work outcomes over the course of treatment was attributable to symptom remission.

This study has several limitations. First, sample selection limits the generalizability. As disability was not measured at trial baseline, this study includes patients who presented with CMD at 2 months and thus were experiencing more chronic episodes of CMD. Moreover, at this follow-up point, 305 patients (10.9%) enrolled in the trial at baseline were missing at 2 months and these missing patients were more likely to be male, younger, and have less severe CMD. The WHODAS assessment did not take place during trial enrollment, during patient primary care visits, in order to minimize the burden on participants. The WHODAS assessments took place at 2 and 6 months, with all other follow-up assessments, during planned visits to participants' homes. Second, the ‘U-shaped’ distribution of the disability days scores, being clustered largely around 0 and 30 days with a small peak at 15 days, suggests recall bias. Because this bias has unpredictable implications for the results, the disability days statistics should be interpreted with caution. Lastly, during piloting the research team observed that participants found the fine judgments required of the 5-point WHODAS scale difficult and thus collapsed the WHODAS scale to three points in the trial. This 3-point response scale did not allow for a fine-grained assessment of responsiveness. In addition, an implication of this collapse of the scale for individual WHODAS items is non-differential misclassification of the WHODAS scores, and thus bias toward the null when assessing correlation and regression coefficients for WHODAS items.

This study provides a foundational step in the development of a common disability metric for evaluating mental health treatment outcomes. The findings suggest that a WHODAS subscale of the six items found to be most strongly associated with CMD severity and recovery could provide a more valid and meaningful measure of CMD recovery in evaluations of CMD interventions. Future research is recommended to confirm the scale reduction of the WHODAS, as evaluated here, by measuring the WHODAS items on a 5-point response scale and using item-response theory methods, such as those used in Bokken (1971). Prior to its use across mental health evaluation studies, the resulting subscale would need to be validated among primary care-seeking populations with CMD in several low- and middle-income countries. The routine use of this CMD-specific subscale of the WHODAS would serve as a critical component of an evidence-based, cost-efficient, and patient-centered response to the challenge of global mental health.
